# Social perception of young adults prolongs the lifespan of aged *Drosophila*

**DOI:** 10.1038/s41514-021-00073-8

**Published:** 2021-09-01

**Authors:** Li-Chun Cho, Chih-Chieh Yu, Chih-Fei Kao

**Affiliations:** 1 Department of Biological Science and Technology, College of Biological Science and Technology, National Yang Ming Chiao Tung University, Hsinchu, Taiwan; 2 Center for Intelligent Drug Systems and Smart Bio-devices (IDS2B), National Yang Ming Chiao Tung University, Hsinchu, Taiwan; 3 Institute of Molecular Medicine and Bioengineering, National Yang Ming Chiao Tung University, Hsinchu, Taiwan

**Keywords:** Ageing, Neuroscience

## Abstract

Lifespan is modulated at distinct levels by multiple factors, including genetic backgrounds, the environment, behavior traits, metabolic status, and more interestingly, sensory perceptions. However, the effects of social perception between individuals living in the same space remain less clear. Here, we used the *Drosophila* model to study the influences of social perception on the lifespan of aged fruit flies. We found the lifespan of aged *Drosophila* is markedly prolonged after being co-housed with young adults of the same gender. Moreover, the changes of lifespan were affected by several experimental contexts: (1) the ratios of aged and young adults co-housed, (2) the chronological ages of two populations, and (3) the integrity of sensory modalities. Together, we hypothesize the chemical/physical stimuli derived from the interacting young adults are capable of interfering with the physiology and behavior of aged flies, ultimately leading to the alteration of lifespan.

## Introduction

Lifespan is modulated by diverse factors that mostly act through the alteration of internal physiology and the initiation of behaviors that respond to the external environment^1–4^. Moreover, the capability to modulate organism lifespan by various genetic and non-genetic manipulations further indicates the plasticity and limitations that apply on the life expectancy of organisms. Intriguingly, recent studies have demonstrated manipulations in the sensory systems, which provide organisms with the ability to perceive and interact with the internal and external environments, are capable of altering organism lifespan, highlighting the potential linkages between sensory perception and aging processes^5,6^. Such an example includes the loss of olfaction, which is the sense of smell that allows organisms to perceive the chemical landscapes of the external environment, significantly influences the lifespan of model organisms, such as worms and fruit flies^7,8^. Extended studies further indicate the plausible connections between the olfactory sensation, the metabolic changes, and ultimately the longevity of organisms^9^. Likewise, increasing evidence suggests the activity of additional sensory modalities also has the capability to differentially influence the physiology and the longevity in animals across taxa (for reviews, see ^5^^, 6^). In the real world, the perception of both external and internal states usually involves multiple sensory-related cells/molecules. Among the distinct perceptions, social perception apprehends social cues emanated from the conspecifics living in the same space and recognizes the changes of social surroundings. For the past years, the influences of social surroundings on the overall health of organisms are well noted from the human-based clinical and epidemiological studies. Both types of studies suggest favorable social experiences are associated with the increase of well-being and healthy aging^6,10^. However, to better understand the physiological impacts of social perception in different social contexts and the underlying neural networks/molecules, small model organisms, such as fruit flies, kept in distinct social situations provide a convenient experimental platform.

Here, we used the model organism, *Drosophila melanogaster*, to explore the physiological impacts to the aged fruit flies when experiencing distinct contexts of social surroundings. More specifically, we focused on the effects conferred by the co-housed young fruit flies. In addition, we further elucidated the sensory pathways in the aged flies that are used to fully perceive the social cues from co-housed young flies and identified the potential components of social cues.

## Results

### The life expectancy of aged flies is differentially affected by the social surroundings

**Fig. 1:**
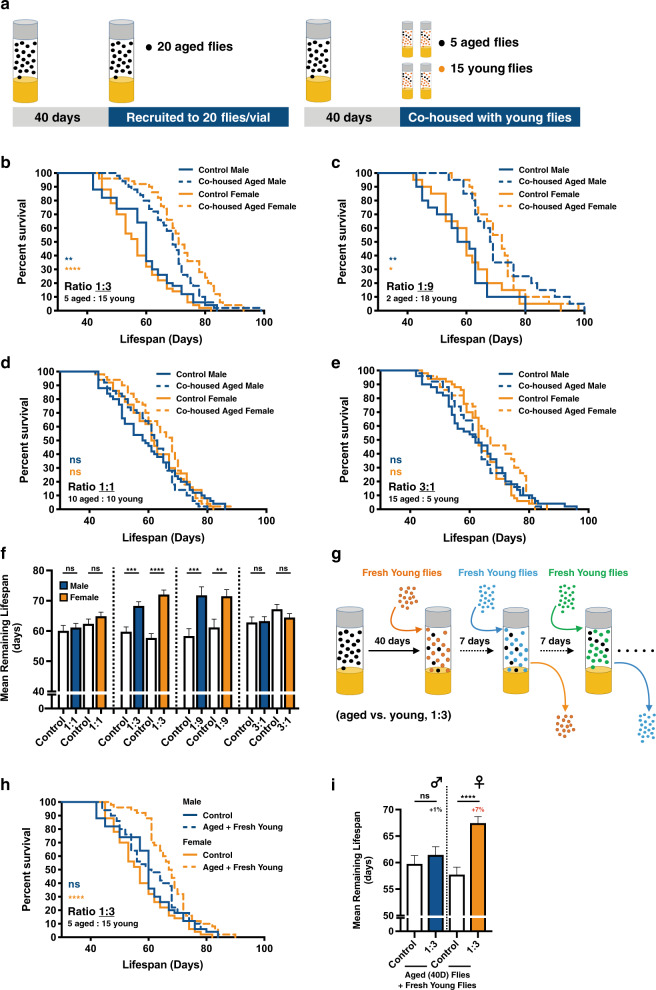
The lifespan of aged flies is altered by co-housing with young adults. **a** An illustration of the co-housing experiments. To keep the population density constant, in the control experiments, the number of 40d-old aged flies was maintained at 20 flies/vial. **b–e** Survival curves of *Canton-S* aged (40d-old, unmated) flies co-housed with young adult flies (1d-old, unmated; same gender) in different cohort ratios (aged:young = (**b**) 1:3; (**c**) 1:9, (**d**) 1:1, (**e**) 3:1). **f** Mean remaining lifespan of the aged flies in different cohort ratios. When co-housed with an exceeding number of young flies, aged flies showed significant lifespan extension (aged: young ratio 1:9, *n* = 20 for each column, samples were collected from 10 independent experimental sets; 1:3, *n* = 50 for each column, samples were collected from 10 independent experimental sets). The mean lifespan of aged flies had no significant changes after being co-housed with an equal or fewer number of young flies (aged: young ratio 1:1 and 3:1; each column contains the results of 50 flies; samples were collected from 5 and 4 independent experimental sets, respectively). Results were expressed as means ± SEM. **g** The experimental scheme of co-housing assays, in which the same five 40d-old *Canton-S* aged flies were co-housed with a fresh batch of 1d-old (15 flies) every 7 days, was illustrated. Lifespan analyses were expressed as (**h**) survival curves and (**i**) means ± SEM. *P* values: ns > 0.1234; <0.0332*; <0.0021**; <0.0002***; <0.0001****. *P* values of the columns were annotated by the Mann**–**Whitney test and survival curves were annotated by the Log-rank (Mantel-Cox) test.

To explore whether the presence of young individuals in the same living environment could affect the lifespan of aged individuals, we used *Drosophila melanogaster* as a model system to create such scenery. Unmated wild-type (WT) flies were cultured according to the standard conditions (see Methods) and were housed at a fixed population size (20 flies/vial). Given the lifespan of fruit flies is profoundly affected by the sex experience/behavior^11–14^, our co-housing experiments only include flies of the same gender to avoid such influences (Fig. 1a). At the age of 40 days (slightly earlier than the median lifespan of *Canton-S* flies; Supplementary Fig. 1a), marked aged flies were relocated to live with 1 day (1d)-old unmated young adults for the rest of their lives. As shown in Fig. 1b, the lifespan of aged *Canton-S* flies was notably affected by co-housing with 1d-old young adults. In our pilot experiments, we kept the ratio of aged vs. young flies at 1:3 to analyze the effects of young adults to aged flies. Intriguingly, in general, the mean lifespan of aged flies showed 14~25% extension compared to controls (Figs. 1b, f; ratio 1:3, aged vs. young; please note that, in the control experiments, the population size of 40d-old flies was kept at 20 flies/vial, same as the experimental groups, to minimize the influences of population density). Similar lifespan extension phenotypes were also observed in two additional WT *Drosophila* strains (Supplementary Figs. 1b**–**1d; *Oregon-R* and *﻿w*^*1118*^; the effects were not significant in *Oregon-R* male flies). Moreover, the lifespan of young flies co-housed was not affected by the presence of aged animals in all three WT fly strains we tested (Supplementary Figs. 3a**–**3b; 1:3, aged vs. young). Increasing the density of young flies in the co-housing experiments further boosted the lifespan extension phenotypes in male flies, but not in female flies (Figs. 1c,f; ratio 1:9, aged vs. young**)**. Overall, regardless of the gender differences, the lifespan of aged fruit flies was prolonged when residing with an exceeding number of young adults. Next, we were interested to know whether the lifespan extension phenotypes persist when an equal amount of aged and young flies were present in the same living environment (Figs. 1d,f; ratio 1:1, aged vs. young). Under this condition, the lifespan of experimental aged flies was comparable to WT flies in both genders. Moreover, in the condition that the density of co-cultured young adult *Drosophila* was decreasing to a 3:1 ratio (aged vs. young), the lifespan of aged female flies was slightly reduced, while aged males were less affected (Figs. 1e,f). Surprisingly, the lifespan of co-housed young flies (at the start of co-housing experiments, they were 1d-old) was significantly reduced by the presence of an equal amount of (1:1, aged vs. young) or an exceeding number of aged flies (3:1, aged vs. young), suggesting the presence of aged flies may bring disadvantages to the survival of co-housed young flies (Supplementary Figs. 3c**–**3e). All together, our results suggest the social environments/interactions have great impacts on the modulation of longevity. However, our following study only focused on how the aged flies respond to the co-housed young adults.

Next, given the overall crowdedness (population size) may have an impact on the organismal lifespan, an additional co-housing experiment was done with a total of 32 (instead of 20) flies in the same culturing vial. The ratio of aged and young flies was also kept the same at 1:3 ratio. As shown in Supplementary Fig. 1e, despite the lifespan extension phenotypes remained, the overall efficacy of pro-longevity effects was not as profound as in the condition of smaller population size (e.g., the mean lifespan of experimental aged female flies showed a 25% increase when culturing in the 20 flies/vial condition, while only 10% increase in the 32 flies/vial condition), arguing the pro-longevity stimuli conferred by the co-housed young flies may be offset by the larger size of the fly population in the confined living space. Together, our results suggest aged flies recognize and respond to the changes in social surroundings, which may directly or indirectly derive from the co-housed unmated 1d-old young fruit flies. We therefore called these longevity-promoting effects, the “youth impacts” (Fig. 1f and Supplementary Fig. 1f). In line with our findings, if the 40d-old aged flies could continue to be surrounded by the fresh batches of 1d-old young flies, was it possible to have stronger longevity-promoting effects (youth impacts) to aged flies? This scenario was achieved by replacing the batch of co-housed 1d-old fruit flies every 7 days (Fig. 1g). Surprisingly, even though the lifespan extension phenotypes remained, the overall effectiveness, regardless of gender, was not as good as the condition that only one batch of young flies was co-housed with the aged flies (Figs. 1h**–**1i, and Supplementary Fig. 1g). The inability of fresh youth impacts to further promote the longevity of aged flies suggests a probable limit of longevity extending capability. Another conceivable explanation highlights the importance of accumulating sufficient strength of social perception to the co-housed young adults. It is likely that aged flies require more time to make beneficial social connections with young flies living in the same space.

**Fig. 2:**
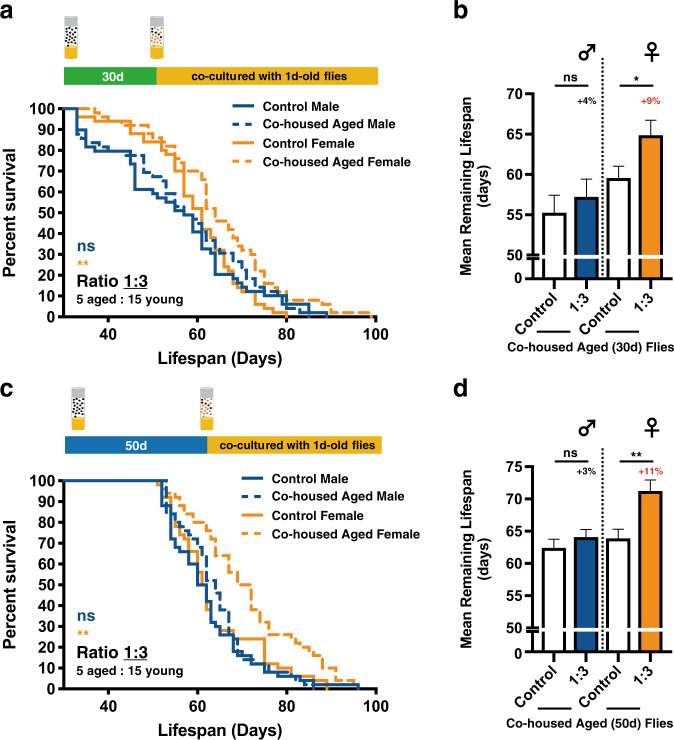
Lifespan extension phenotypes are affected by the chronological age of aged flies. *Canton-S* flies of indicated chronological ages (30d- and 50d-old) were co-housed with 1d-old flies at the cohort ratio of 1:3. Results were expressed as (**a**, **c**) survival curves and (**b**, **d**) means ± SEM. In all cases, the longevity-promoting effects were more robust in female flies. *P* values: ns > 0.1234; <0.0332*; <0.0021**. *P* values of the columns were annotated by the Mann**–**Whitney test and survival curves were annotated by the Log-rank (Mantel-Cox) test. Sample size: *n* = 49~50 for each column; results were collected from 10 independent experimental sets.

**Fig. 3:**
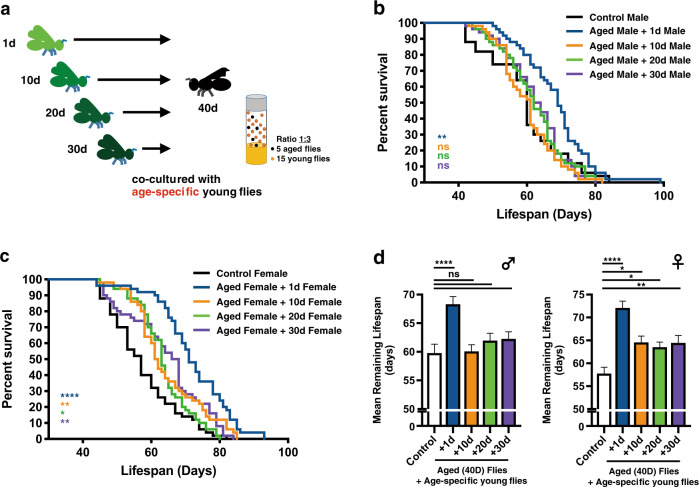
Chronological ages of the co-housed young flies have different longevity-promoting capabilities. **a** An illustration of the experimental concept, in which aged flies were co-cultured with age-specific young flies, was delineated. Briefly, 40d-old *Canton-S* aged flies were co-housed with 1d-, 10d-, 20d-, and 30d-old young flies respectively. Results were expressed as survival curves (**b**) for male flies, and (**c**) for female flies) and (**d**) means ± SEM. In all conditions, aged flies acquired stronger lifespan extension phenotypes when co-housed with younger flies. *P* values: ns > 0.1234; <0.0332*; <0.0021**; <0.0002***; <0.0001****. *P* values of the columns were annotated by the Kruskal–Wallis test and followed by Dunn’s multiple comparison test and survival curves were annotated by the Log-rank (Mantel-Cox) test. Sample size: *n* = 50 for each column; results were collected from 10 independent experimental sets.

Next, to study if the youth impacts were also effective on aged flies that are slightly younger/older than 40 days, similar co-housing experiments were performed accordingly. While similar lifespan extension phenotypes were observed, the overall effects on the 40d-old adult *Drosophila* were more profound in comparison to 30d- and 50d-old flies (Fig. 2 and Supplementary Fig. 2). These results suggest there may be a best chronological window for the aging flies to receive the youth impacts from co-housed young adults. However, it is currently not clear why 40d-old fruit flies have the utmost responses to the youth impacts. The following interesting question we asked was what chronological ages of young flies are able to confer the pro-longevity signals. To this end, 40d-old flies were arranged to live with the same gender, unmated flies of selected chronological ages younger than 40 days (Fig. 3a; 1d-, 10d-, 20d-, and 30d-old flies). Our results showed that aged flies perceive the most notable lifespan promoting signals from the youngest flies (i.e., co-housing of 40d-old and 1d-old flies; the age difference is 39 days; Figs. 3b**–**3d). Strikingly, the lifespan extension phenotypes were almost completely lost in 40d-old male flies that were co-housed with 10d-old unmated males, as well as 20d- and 30d-old adults (Fig. 3b). However, unlike the male flies, aged female flies appeared to be less sensitive to the chronological ages of co-housed younger females. In all conditions we tested, the lifespans of aged female flies were readily prolonged, albeit the effects were less potent (Fig. 3c). In summary, our results suggest the deterioration of youth impacts occurs quickly within less than 10 days (Fig. 3d and Supplementary Fig. 4).

### Aged flies that are co-housed with young adults display better physiological fitness

**Fig. 4:**
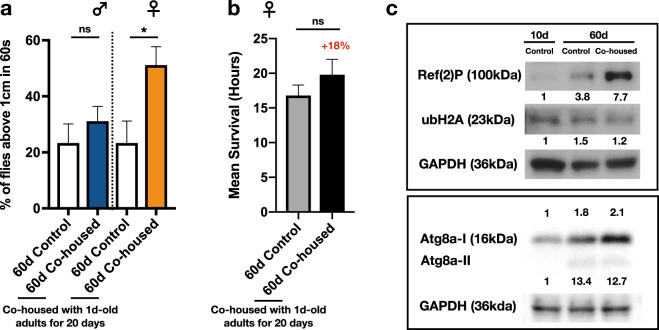
Aged flies receiving the youth impacts maintain better physiological fitness. **a** Motor activity (negative geotaxis) was assayed in the 60d-old control *Canton-S* flies (indicated as 60d Control) and 60d-old aged flies that have been co-housed with 1d-old young *Canton-S* flies for 20 days (indicated as 60d Co-housed). Each condition included 6 independent replicates and a total of 30 flies. Results were expressed as mean percentages ± SEM. **b** 60d-old control *Canton-S* flies (indicated as 60d Control) and 60d-old aged flies that have been co-housed with 1d-old young *Canton-S* flies for 20 days (indicated as 60d Co-housed) were subjected to desiccation. The mean survival time ± SEM of each condition was calculated and illustrated as the bar graph. *P* values: ns > 0.1234; <0.0332*. *P* values of the columns were annotated by the Mann**–**Whitney test. Each condition included 10 flies. **c** Protein levels of Ref. ^2^ P, Atg8a-I/Atg8a-II, ubH2A, and GAPDH in the whole animal lysates derived from flies of the indicated condition were examined by the Western blot analyses. Label of each lane was: (1) 10d, Control: 10d-old young *Canton-S* flies. (2) 60d, Control: 60d-old aged *Canton-S* flies, (3) 60d, Co-housed: 60-old aged *Canton-S* flies that have been co-housed with 1d-old young flies for 20 days. Levels of GAPDH were used to normalize total protein input. Each lane contained whole animal lysates from 5 flies. Results from 2 independent experiments (except the case of Atg8a, triplicates were included) were used to quantitate the differences of protein expression (via the ImageJ program). Fold changes of expression level were compared to the value of 10d, Control (the very left lane; assigned as 1) and indicated at the bottom of Western blot. All Western blots derived from the same experiment were processed in parallel.

Despite the lifespan extension phenotypes were robust, it remained to be elucidated whether the youth impacts also confer the physiological fitness to aged flies. To this end, two simple behavioral assays were adopted to evaluate the physical fitness of aged flies receiving the youth impacts. After co-housing with the 1d-old young adults (1:3; aged vs. young) for 20 days, the aged (now 60d-old) fruit flies were subjected to the fly climbing assay (negative geotaxis assay). As shown in Fig. 4a, the aged flies receiving the youth impacts had much better motor performance than the age-matched control flies. Similar to the lifespan extension phenotypes, the increase of motor performance was more profound in the aged female flies. Moreover, the desiccation resistance was also significantly better in aged flies that were co-housed with young adults for 20 days (Fig. 4b). Taken together, by receiving the youth impacts for 20 days, the physical conditions, such as the deterioration of motor activity and the decline of stress resistance, were markedly alleviated, suggesting the youth impacts are able to extend the healthspan of aged flies.

Besides remaining physically active during the extended lifespan, we also like to know the potential changes of underlying molecular features, especially factors known to exhibit pro-longevity capability, such as the activation of autophagic activity^15–18^. Based on the earlier studies, enhancing the activity of autophagy by ectopic expression of Ref. ^2^ P or Atg8a in flies is able to promote the longevity^16,19^. Interestingly, as evident by the immunostaining, protein levels of Ref. ^2^ P and Atg8a were both substantially up-regulated in the 60d-old aged flies receiving the youth impacts (Fig. 4c). Moreover, consistent with the earlier studies, we also noted the autophagic activity is activated in the control aged flies, albeit slightly weaker than the experimental aged flies. Given that autophagy has been demonstrated predominantly cytoprotective in multiple models^20,21^, our results therefore suggest the pro-longevity effects of youth impacts may operate by triggering stronger autophagic activity in the aged flies. Furthermore, a recent study by Yang et al.^22^ has demonstrated the age-dependent increase of ubiquitinated histone 2 A (ubH2A) as a conserved aging marker across different species, including *Drosophila*. As shown in Fig. 4c, the aged control flies had slightly higher levels of ubH2A than the 10d-old young adults. Intriguingly, the ubH2A levels in the aged flies after co-housing with 1d-old young adults for 20 days were similar to that of 10d-old control flies, suggesting the aged flies receiving the youth impacts may retain the physiological condition of younger fruit flies. In summary, our results of behavioral assays and the molecular features associated with pro-longevity suggest the youth impacts are capable of extending the healthspan of aged flies.

### Social perception involves the olfactory and gustatory systems

**Fig. 5:**
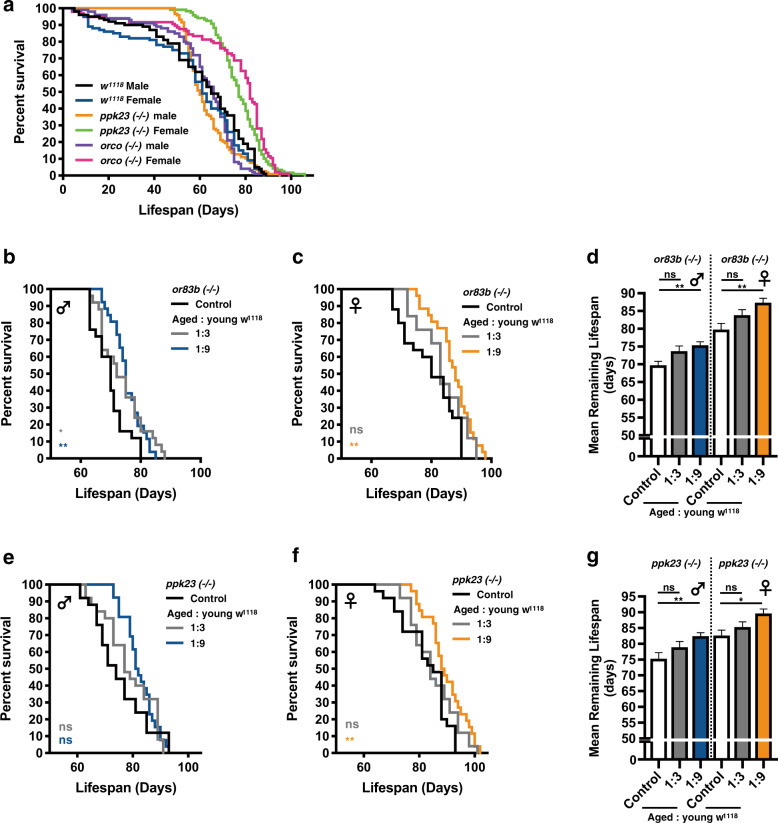
Social perception of co-housed young flies by the aged flies involves the sensory inputs from olfaction and gustation. **a** Survival curves of the *or83b(−/−)*, *ppk23(−/−)*, and *w﻿*^*1118*^ control flies were shown. All flies were maintained at the density of 20 flies per vial. *n* = 96–119 in each genotype/gender. Lifespan results of aged *or83b* **(b–d)** or *ppk23* **(e–g)** mutant flies co-housed with young *w*^*1118*^ at the ratio of 1:3 and 1:9 (aged: young) were expressed as survival curves and means ± SEM. *P* values: ns > 0.1234; <0.0332*; <0.0021**. *P* values of the columns were annotated by the Kruskal–Wallis test and followed by Dunn’s multiple comparison test and survival curves were annotated by the Log-rank (Mantel-Cox) test. Sample size: (1) *n* = 25 for the control flies, samples were randomly collected from 3 independent experimental sets, (2) *n* = 25 for the co-housing experiments performed at 1:3 ratio; results were collected from 5 independent experimental sets, (3) *n* = 26 for the co-housing experiments performed at 1:9 ratio; results were collected from 13 independent experimental sets.

In considering the olfactory sensation, which mediates the detection and recognition of volatile compounds, is one of the most important sensations in insects^23–25^, we were interested to know whether olfaction is involved in perceiving the youth impacts. Flies with defective *or83b* gene are anosmic and live longer, especially in the mutant female flies (Fig. 5a and Supplementary Fig. 5a)^8,26,27^. Given the longer lifespan of *or83b* mutant flies, we included the 60d-old, but not the 40d-old, mutants in the co-housing experiments. As shown in Figs 5b–d, the lifespan of aged *or83b* mutants was not significantly extended after being co-housed with 1d-old WT adults at the 1:3 ratio. Our results therefore suggest olfaction may be involved in the perception of youth impacts. However, in addition to the olfaction, the gustation also provides another spectrum of environmental cues to the organisms, such as pheromones and trace of foods^28–30^. Different from the mechanisms of olfaction, it is experimentally challenging to eradicate gustatory perception entirely due to the presence of diverse gustation-related receptors and lack of common regulatory mechanism^31^. Physical ablation of the labellum, legs, and wings, which house most of the gustatory neurons, dramatically weakened the flies, leading to organism death within a few days. We therefore selectively tested the *ppk23* (pickpocket 23) mutants in the co-housing experiments. Ppk23, a member of the Degenerin/Epithelial sodium channel (DEG/ENaC) family, is a gustatory-specific putative non-voltage gated cation channel and is broadly expressed in taste-related neurons^32–35^. The survival assays indicated the lifespan of *ppk23*^−/−^ female flies, but not male flies, was longer than *w﻿*^*1118*^ controls (Fig. 5a and Supplementary Fig. 5a). Therefore, similar to the *or83b* mutants, 60d-old *ppk23*^*−/−*^ flies were assayed in the co-housing experiments. As shown in Figs. 5e**–**g, we surprisingly found the lifespan of aged *ppk23* (60d-old) mutants was also not significantly extended after being co-housed with the 1d-old *w*^*1118*^ adults at the 1:3 ratio. Therefore, our results suggest the functional integrity of both olfaction and gustation may be critical for aged flies to perceive and recognize the youth impacts. However, it was curious whether the increased density of co-housed young adults (i.e., ratio 1:9, aged vs. young) is able to confer the pro-longevity effects on either *or83b* or *ppk23* mutant flies. Under such co-housing conditions (Figs. 5b**–**g; ratio 1:9), the efficacy of youth impacts on either aged *or83b* or *ppk23* mutant flies appeared to be much stronger, as evident by the extended lifespan. Together, these results further suggest loss of either sensory modality (olfaction or gustation) led to the impaired ability to fully appreciate the changes of social surroundings (Supplementary Fig. 5b).

**Fig. 6:**
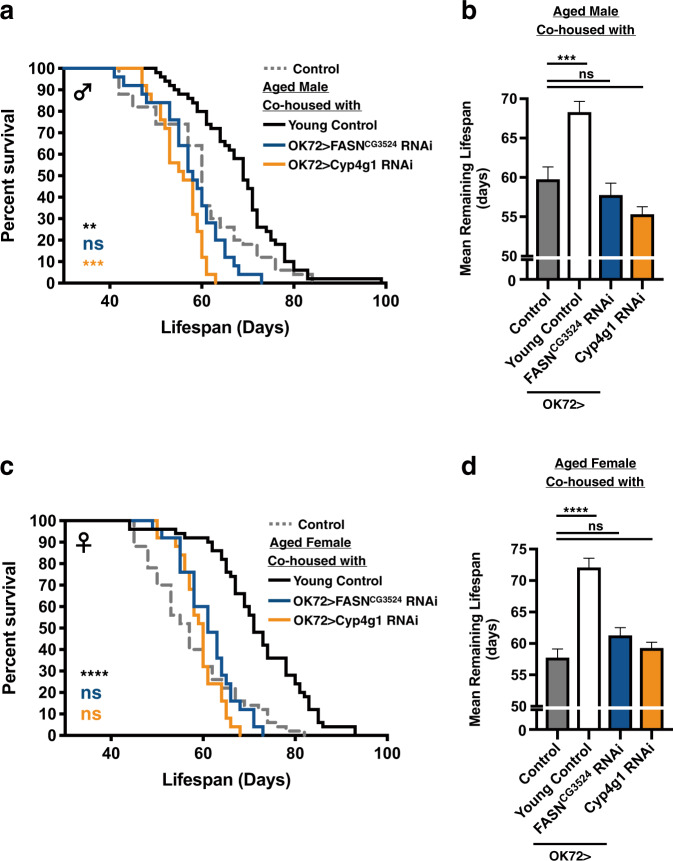
The pro-longevity capability is diminished in young flies defective in generating cuticular hydrocarbons (CHCs). 40d-old aged flies were co-housed with 1d-old young control (indicated as Young Control), OK72-GAL4 > UAS-FASN^CG3524^ RNAi (indicated as OK72 > FASN^CG3524^ RNAi), and OK72-GAL4 > UAS-Cyp4g1 RNAi (indicated as OK72 > Cyp4g1 RNAi) flies at the ratio of 1:3. Survival results of co-housed aged flies were expressed as survival curves and means ± SEM. (**a**, **b**) for male flies, and (**c**, **d**) for female flies. *P* values: ns > 0.1234; < 0.0021**; < 0.0002***; <0.0001****. *P* values of the columns were annotated by the Kruskal–Wallis test and followed by Dunn’s multiple comparison test and survival curves were annotated by the Log-rank (Mantel-Cox) test. Sample size: (1) Control and Young Control, *n* = 50 from 10 independent experimental sets, (2) OK72 > FASN^CG3524^ RNAi and OK72 > Cyp4g1 RNAi, *n* = 25 from 5 independent experimental sets.

Next, to explore the identity of the youth impacts, we collected the cuticular extracts (including cuticular hydrocarbons, pheromones, and other organic chemicals that were dissolved by the hexane solution) from 1~2d-old young flies, since social interaction between flies mostly involves chemical communications^35–38^. To rid of other external sources of cuticular chemicals, only a single fly was cultured in a space-restricted vial containing a piece of cuticular extract-rinsed filter paper (Supplementary Fig. 6a). Reduction of living space was to increase the frequency of contact to the cuticular extracts. Moreover, to maintain the efficacy of cuticular extracts, the fly was transferred to a new vial with fresh cuticular extracts every two days. As suspected, the exposure to hexane alone reduced the lifespan of adult flies (+Hexane) when comparing to mock controls (Control; Supplementary Fig. 6b). However, flies living in the cuticular extract-conditioned vial had a slightly longer lifespan than the hexane-only group. Overall, the pro-longevity effects were more robust in male flies, suggesting the strength of youth impacts may partly derive from chemical signals transferred from the co-housed young flies. However, the low efficacy of cuticular extracts may result from the low frequency of physical contact with the filter paper or the insufficient amount of cuticular extracts applied. Moreover, the cuticular extracts isolated by the organic solvent may not represent all the chemicals that could be found on the body of fruit flies, since lots of the cuticular chemicals are volatile and have a relatively short half-life. Nonetheless, another approach to explore the functional contribution of cuticular hydrocarbons (CHCs) to the youth impacts is to alter the CHC profile of co-housed young flies. Two recent studies have demonstrated the generation of CHCs can be markedly suppressed by silencing the expression of FASN^CG3524^ (fatty acid synthase) or Cyp4g1 (cytochrome P450-4g1) via RNAi in the fly oenocytes^39,40^. Given that both FASN^CG3524^ and Cyp4g1 are enzymes required for the biosynthesis of CHCs, flies with reduced levels of FASN^CG3524^ or Cyp4g1 display very different CHC profiles. Consistent with our hypothesis, the longevity of aged flies was not promoted by co-housing with either CHC-deficient flies (Fig. 6 and Supplementary Fig. 7; OK72-GAL4 > UAS-FASN^CG3524^ RNAi flies or OK72-GAL4 > UAS-Cyp4g1 RNAi flies). Moreover, we also noted the aged male flies were more sensitive to changes of CHC profiles, as evident by the shorter lifespan when comparing to the control flies (Fig. 6b). However, the causes remain to be studied. Taken together, our results suggest the chemical signals from CHCs are important players of the pro-longevity effects conferred by the youth impacts. Disturbing the CHC metabolism may markedly interfere with the efficacy of youth impacts. Furthermore, in addition to the chemical communications, it is also possible that physical engagement with the young flies may partly contribute to the effects of youth impacts.

## Discussion

Besides extending healthspan, the youth impacts may provide additional beneficial effects to various disease conditions. For example, a previous study has demonstrated social interactions with co-housed young flies are able to improve the physiological defects, such as the reduction in lifespan, stress resistance, and motor activity, displayed in the SOD (superoxide dismutase) mutant flies^41^. Another interesting study also indicates the heterogeneity of the social environment mediates the cancer progression in the *Drosophila* model^42,43^. Consistent with their discoveries, the lifespan of fruit flies living in the same space was affected by the compositions of aged and young adults (Fig. 1). In this study, we first demonstrated the existence of “youth impacts” and their capability to promote the increase of lifespan in aged flies. To benefit from the youth impacts, the number of co-housed young flies in the same living space had to be greater than the aged flies (e.g., Fig. 1, ratio 1:3 and 1:9). Presumably, the signals of youth impacts have to be sufficiently large enough to initiate the pro-longevity effects in the aged flies. Furthermore, to perceive the most beneficial effects of youth impacts, there was a best chronological window for aged flies to make such social connections with young flies. As indicated in Figs. 1 and 2, the lifespan-extending effect of youth impacts was most profound in flies of 40d-old, but not in flies of younger or older ages. These results suggest the sensory systems within the 40d-old flies may be particularly responsive to the youth impacts. Also notably, the youth impacts were expired very soon after 10 days of adult life (Fig. 3). For the aged flies to perceive the full effects of youth impacts, the sensory inputs from olfaction and gustation were essential (Fig. 5). Therefore, the functional integrity of olfactory and gustatory sensing systems in the aged flies may determine the efficacy of youth impacts. Consistently, diverse sensory modalities and associated neural circuits have been implicated in modulating the energy homeostasis and longevity of organisms^5,6^. Lastly, in considering the experimental setups we used in this study, one general limitation was the constant cohort density at the beginning of co-housing experiments (Fig. 1a). This experimental design is to minimize the influences of population density on the lifespan of co-housed fruit flies. Therefore, the effects of young adult flies to the aged fruit flies could only be assessed in selected co-housing conditions (such as the selected cohort ratios shown in Fig. 1).

Based on our results, the most profound effects of longevity promotion by the youth impacts were around 14 to 25% of the increase (Fig. 1). Comparing to the efficacy of known pro-longevity manipulations^44–46^, the overall effects provided by youth impacts were modest. However, similar to the lifespan extension mediated by the reduction of caloric intake^47,48^, the elicitation of youth impacts does not involve the manipulation of endogenous/ectopic genetic element(s) or drug treatment. Instead, the youth impacts work through the variations of social surroundings and the contents of social engagement, representing a more natural pro-longevity approach. Furthermore, multiple lines of evidence suggest the social perception mechanisms are mostly mediated by smell- and taste-dependent chemical cues, such as pheromones and additional volatile/non-volatile chemicals found on the fly body. Consistently, the young CHC-deficient flies were not able to confer the youth impacts to aged fruit flies (Fig. 6). More intriguingly, earlier studies have demonstrated the chemical compositions of know pheromones vary significantly in fruit flies of distinct chronological ages^6,36^. Therefore, the unique pheromone profiles of young fruit flies may act as an important source of youth impacts and the sensory perception to these chemicals may directly or indirectly influence the physiology and aging process of aged flies. At present, it is still not clear if the youth impacts are effective between different *Drosophila* species, given the pheromone compositions show distinctive features among *Drosophila* species^49,50^. In considering the potential social cues, the human body odors are found to be able to convey a variety of biological and social information to the surrounding individuals. Comparable to the cuticular substances found on the body of fruit flies, the chemical compositions of human body odor are also extremely complicated and change in an age-dependent manner^51,52^. One such example is the increase of 2-Nonenal (C_9_H_16_O), an unsaturated aldehyde with an unpleasant greasy and grassy odor, in the body of elders, suggesting its involvement of the age-related change of body odor^53^. Also consistently, a previous study has confirmed the connection between social network size and olfactory function^54,55^. Future studies to identify and characterize chemicals from the scents emanated from young organisms (including the young fruit flies) that possess beneficial/pro-longevity capability will help us uncover the mechanisms of youth impacts and roles of social perception.

It is known that sensory perception has direct influences on physiology and aging progression^5,6,41^. Animals not only passively experience the changes of diverse external and internal states, but also evaluate and react according to the changes. The key internal states include the concentrations of metabolites, the energy balance, and the osmolarity of body fluid. On the other hand, the perceptive experiences to social surroundings, time, and potential stresses/threats, provide the links of external environments to internal physiology and neural processing. Particularly, many clinical studies have also associated favorable social surroundings with the increase of overall well-being and the reduction of distinct aging-related diseases^10,56,57^. Here, our results using the *Drosophila* model system demonstrated the longevity-promoting capability of youth impacts derived from young flies of the same gender. Albeit the effects of youth impacts from the opposite gender are not tested in this study, earlier studies indicate the sexual activities and functional fertility could incur a survival cost^6,58–60^. However, intriguingly, successful copulation can endow the male flies with beneficial effects^6^, suggesting sex behaviors/experiences may differentially influence the life expectancy of male and female flies.

The definition of healthy aging is to allow elderly individuals to maintain proper functions in physiology, mental status, and social well-being. In addition to the advances of medical care and disease prevention, in modern human society, studies by social scientists also suggest cross-generational co-housing, which brings active social engagements between elders and younger adults, is a promising and viable approach to promote healthy aging of elders, highlighting the influences of social surroundings^61,62^. Here, our study in the *Drosophila* model provided extensive experimental evidence that not only substantiates the notion that social perception/interaction has a great impact on the healthspan of aged individuals, but also points out potential limitations and requirements for the best efficacy of youth impacts.

## Methods

### *Drosophila* strains

Flies were reared with standard cornmeal-yeast-agar medium at 25 °C, and on a 12/12 hr light/dark cycle. The following *Drosophila* lines were used: *w*^*1118*^ (BDSC 3605), *Canton-S* (BDSC 64349), *Oregon-R* (a gift from Dr. Chun-Hong Chen), *or83b* mutant (BDSC 23130; w[*]; TI{w[+m*]=TI}Orco[2]), *ppk23* mutant (BDSC 12571; w[1118]P{w[+mGT]=GT1}ppk23[BG01654]), OK72-Gal4 (BDSC 6486), UAS-FASN^CG3524^ RNAi (VDRC 4290), and UAS-Cyp4g1 RNAi (VDRC 110678).

### Lifespan assay

Unmated flies were collected and separated by gender soon after eclosion. 20 or 32 flies were sorted in a vial and incubated at 25 °C. Flies were transferred to a new vial containing fresh medium every 2–3 days. The number of dead flies was counted and recorded. Survival analyses were performed using the Prism 7 (GraphPad Software). To explore the effects of cuticular extracts, a single fly was cultured in a vial with a filter paper (1 cm^2^) rinsed with isolated cuticular extracts from three 1d-old young flies. For the lifespan of co-housing experiments, please see the detailed procedure below. For the survival analysis under desiccation, ten 60d-old flies (each set of experiments) were relocated into an empty vial. The death of the flies was counted every 6 h.

### Co-housing experiments

Unmated flies were collected and sorted into 20 or 32 flies per vial soon after eclosion. After 2–3 days, the right-wing of flies was clipped to remove the portion beyond the rear end of the abdomen under CO_2_ anesthetization. The marked flies were used as the experimental aged flies. At the indicated dates, aged flies and unmated young flies were cultured in the same vial at the specified ratio (aged: young; 1:9, 1:3, 1:1, 3:1) and keep the population density at 20 flies/vial. For 32 flies/vial, flies were cultured at the ratio of 1:3 (aged: young). Death of co-housed aged and young flies were counted and recorded every 2–3 days. For the controls of the co-housing experiments, the age-matched siblings were recruited to the control vial at the indicated dates to keep the same population density at 20 or 32 flies/vial. Deaths of control flies were recorded along with the experimental groups. Statistic analyses were performed with the Prism 7 software (GraphPad Software).

### Collection of cuticular extracts

Fifteen 1d- to 2d-old young flies were collected and soaked into hexane (10 μL per fly; Sigma #32293). After 1 h of incubation on the rocking shaker at room temperature, hexane was removed by the water circulating vacuum pump. The dried cuticular extracts were stored at −20 °C. Right before the experiments, the cuticular extracts were resolved with 30 μl hexane and applied onto a 1 cm × 1 cm filter paper (Whatman #1001-090). The filter paper was then placed into the vial containing fresh fly food. A single fly was introduced into the conditioned vial and transferred to a new vial every 2 days. Also note, during the assay, the living space of the single-housed fly was reduced to half by adjusting the height of the cotton stopper. Restriction of the living space was to increase the frequency of physical contact with the filter paper.

### Measurement of motor activity

Briefly, five flies were placed into an empty vial without anesthesia. After acclimating for 30 min at room temperature, flies were tapped to the bottom of the tube. The number of flies that reached over 1 cm in 60 s was counted and recorded. For each set of assay (5 flies), three trials were performed. Each condition included 6 independent replicates. Results were presented as mean percentages ± SEM.

### Antibodies

Primary antibodies specific to the following substrates were used: Ref. ^2^ P (1:3000, #178440, Abcam); ubH2A (1:5000, #8240S, Cell Signaling Technology); Atg8a (1:1000; a gift from Dr. Guang-Chao Chen and Dr. Horng-Dar Wang); and GAPDH (1:10,000, #627408 and #100118; GeneTex). Goat anti-rabbit IgG (HRP) was used as the secondary antibody in the Western Blot analyses (1:10,000, #213110-01, GeneTex).

### Western blot

Female flies were homogenized with a mixture of RIPA buffer and sample loading buffer. The samples were then denatured for 10 min at 98 °C. An equal amount of proteins were subjected to electrophoresis on a 10% acrylamide gel and transferred onto a polyvinylidene difluoride membrane. After 1 h blocking with 5% skim milk in Tris Buffered Saline with Tween-20 (TBST) at room temperature, membranes were incubated with the primary antibodies at 4 °C overnight. After washing, the membranes were incubated with HRP-conjugated secondary antibody (1:10,000) for 1 h. The protein bands were detected with enhanced chemiluminescence (ECL) Western blotting detection reagent (Millipore, Burlington, MA, USA).

### Quantification and statistical analysis

Statistical tests were conducted using Prism 7 (GraphPad Software). The lifespan of aged flies was plotted as columns with mean lifespan ± SEM and the differences were evaluated by the Mann–Whitney test. For multiple lifespan comparisons, the differences were evaluated by Kruskal–Wallis test followed by Dunn’s multiple comparison test. The survival curves were plotted as Kaplan Meyer plots and the statistical significance was tested using the log-rank (Mantel-Cox) test.

### Reporting summary

Further information on research design is available in the Nature Research Reporting Summary linked to this article.

## Supplementary information

Supplementary Information

Reporting Summary

## Data Availability

All data are available in the paper or the supplementary materials; raw data are available upon request.
