# Supplementary material for: Social perception of young adults prolongs the lifespan of aged Drosophila
Source: NPJ Aging Mech Dis. 2021 Sep 1;7:21. doi: 10.1038/s41514-021-00073-8 (PMC8410773; doi:10.1038/s41514-021-00073-8)

Supplementary Materials for

Social Perception of Young Adults Prolongs the Lifespan of Aged Drosophila

Li-Chun Cho[1,#], Chih-Chieh Yu[2,3,#], Chih-Fei Kao[1,2,*]
Correspondence to: kcfei@nctu.edu.tw

This PDF file includes:

Supplemental Figures 1 to 7
Raw images of Western blots (Ref(2)P, ubH2A, Atg8a-I/II, and GAPDH)

Supplemental Figures

Supplemental Fig. 1. The youth impacts can be observed in w^{1118} and Oregon-R flies

(a) Survival curves of the Canton-S flies. Flies were kept at the density of 20 flies per vial and their survival was recorded every two days. The median lifespan of Canton-S was about 62 days in male flies (n=189) and 60 days in female flies (n=184). **(b)** 40d-old Oregon-R or **(c)** w^{1118} flies were Co-housed with 1d-old young flies at the ratio of 1:3. The lifespan of individual flies was recorded and analyzed. In both WT lines, the mean lifespan of aged flies was notably increased to different levels, except the case of Oregon-R male flies. Results were expressed as means ±SEM. Each column contains results of 50 flies from 10 independent assays. **(d)** Summary of results shown in (Supplementary Fig. 1b-1c). **(e)** Survival analysis of 40d-old Canton-S flies co-housed with 1d-old young flies at the ratio of 1:3 (8 aged vs. 24 young). Columns were expressed as means ±SEM. **(f)** Summary of results shown in Figs. 1b-1f. **(g)** Summary of results shown in Figs. 1h-1i. P values: ns>0.1234; <0.0021 **; <0.0002 ***; **** <0.0001. p values of the columns were annotated by the Mann-Whitney test. p values of the survival curves were annotated by the Log-rank (Mantel-Cox) test.

Supplemental Fig. 2. Survival analyses of 30d- and 50d-old flies that are co-housed with young flies

Summary of results shown in Figs. 2a-2d.

Supplemental Fig. 3. The lifespan of experimental co-housed young flies is not affected by the aged flies

(a) Survival curves of the experimental 1d-old young flies (Exp) (three WT species were shown: Canton-S, Oregon-R, and w^{1118}) that were co-housed with 40d-old aged flies at the 1:3 ratio. There is no significant differences between the Exp flies and the control flies (Cont; 20 flies/vial). p values were annotated by the Log-rank (Mantel-Cox) test. In all cases, p values were greater than 0.05. **(b)**

Summary of results shown in (a). **(c and d)** Survival curves of Canton-S 1d-old young flies that were co-housed with 40d-old aged flies at the ratio of 1:9, 1:3, 1:1, and 3:1. Columns were expressed as means ±SEM. P values: ns>0.1234; <0.0332 *; <0.0021 **; <0.0002 ***. P values of the columns were annotated by the Kruskal-Wallis test and followed by Dunn's multiple comparison test. P values of the survival curves were annotated by the Log-rank (Mantel-Cox) test. **(e)** Summary of results shown in (c and d).

Supplemental Fig. 4. The lifespan extension phenotype is observed in the aged flies that are co-housed with
flies of selected chronological ages

Summary of results shown in Figs. 3b-3d.

Supplemental Fig. 5. Results of co-housing experiments using aged *or83b* and *ppk23* mutant flies

(a) Summary of results shown in Fig. 5A. **(b)** Summary of results shown in Figs. 5b-5g.

Supplemental Fig. 6. Explore the lifespan modulatory effects of cuticular extracts collected from young flies

(a) The experimental illustration describes the processes of retrieving cuticular extracts from 1~2d-old Canton-S flies and the subsequent culturing conditions. **(b)** To explore the potential source of youth impacts conferred by the young flies, a single Canton-S fly was cultured in a vial containing the regular cornmeal food and a piece of filter paper rinsed with cuticular extracts isolated from three 1d to 2d-old same gender flies (+ Cuticular extracts). The fly was transferred to another vial containing cornmeal food and the freshly prepared cuticular extract-rinsed filter paper every two days. For the hexane control experiments, a single fly was cultured with the filter paper rinsed with hexane (+ Hexane). Another control experiment included only a single Canton-S fly without any

treatment (Control). Results were expressed as means ±SEM. P values were annotated by the Kruskal-Wallis test and followed by Dunn's multiple comparison test (each column, n=10). P values: ns>0.1234; <0.0332 *; <0.0002 ***.

Supplemental Fig. 7. Co-housing experiments using the young CHC-deficient flies

Summary of results shown in Figs. 6a-6d.

Supplementary Figure 1

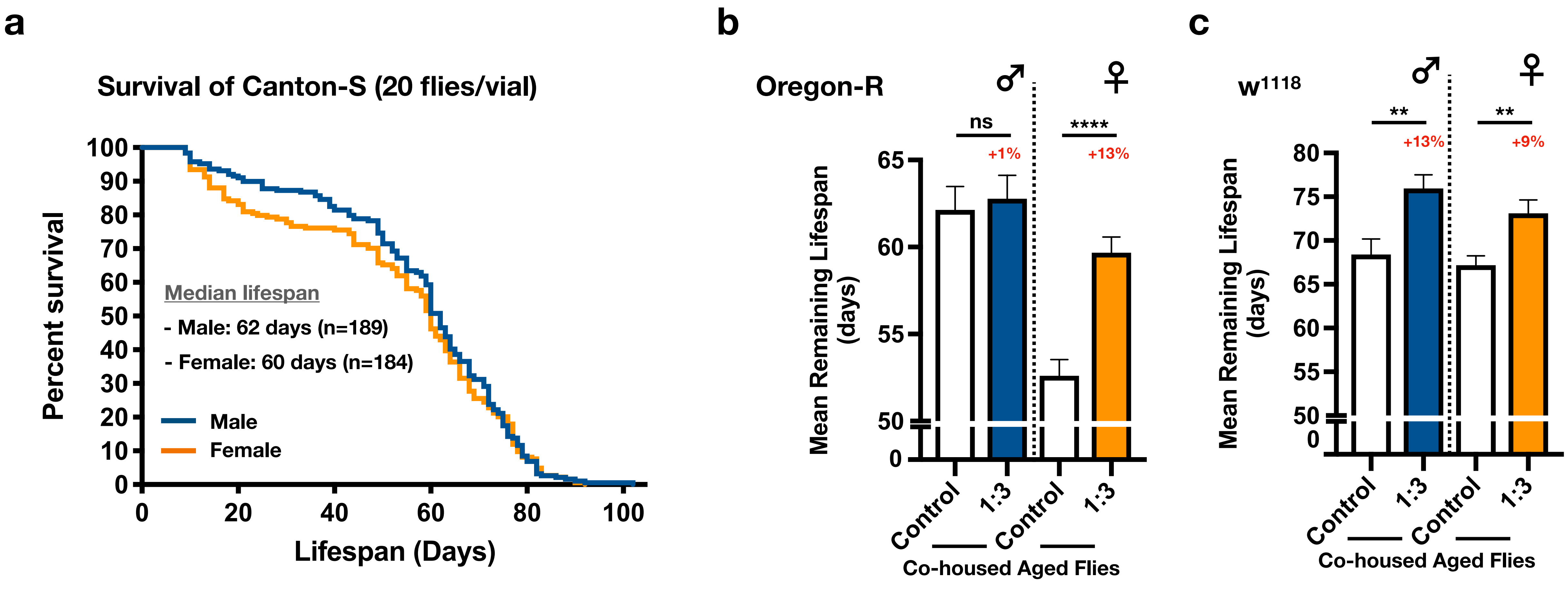

d

Mean Lifespan (Days) ± SEM	Control	n=	Co-housed Aged (40D) Flies	n=
**Male**				
Canton-S	59.76 ± 1.58	50	68.30 ± 1.36	50
Oregon-R	62.14 ± 1.34	50	62.78 ± 1.35	50
w^{1118}	68.40 ± 1.77	60	75.94 ± 1.57	50
**Female**				
Canton-S	57.74 ± 1.39	50	72.08 ± 1.49	50
Oregon-R	52.60 ± 0.93	50	59.68 ± 0.90	50
w^{1118}	67.18 ± 1.07	60	73.10 ± 1.52	50

Supplementary Figure 1

e

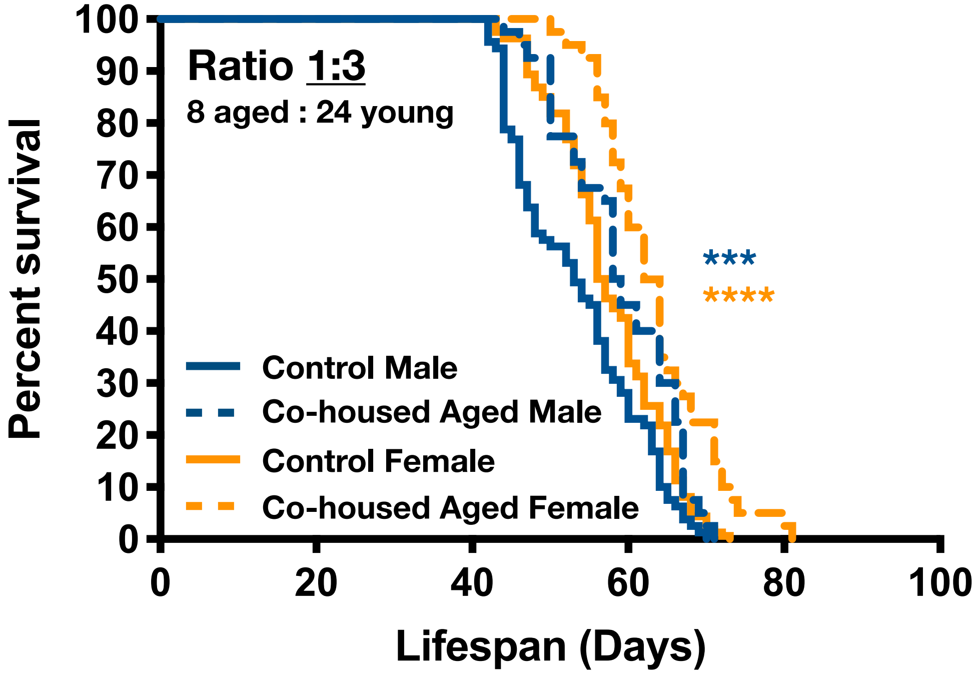

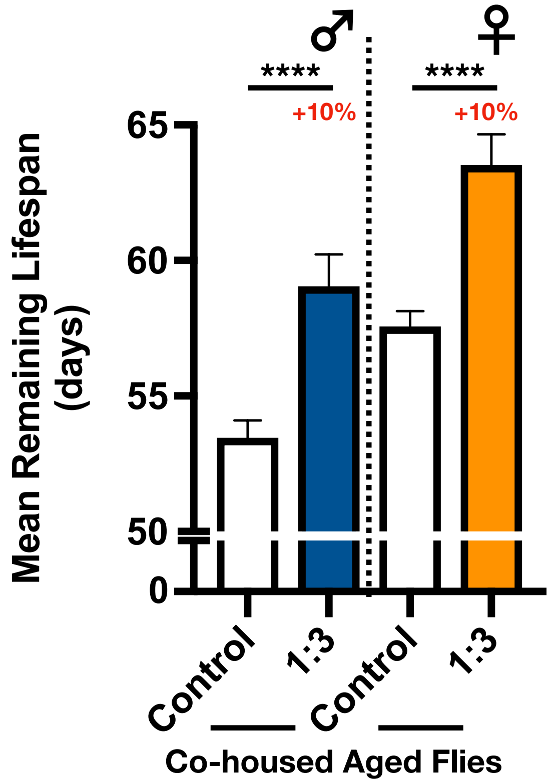

Ratio 1:3<br>8 aged : 24 young	Control			Co-housed Aged (40D) Flies		
	Median lifespan (Days)	Mean Lifespan (Days) ± SEM	n=	Median lifespan (Days)	Mean Lifespan (Days) ± SEM	n=
**Male**	53	53.46 ± 0.65	160	58.5	59.05 ± 1.18	40
**Female**	56.5	57.58 ± 0.56	160	63	63.53 ± 1.13	40

Supplementary Figure 1

f

	Median lifespan (Days)	Mean Lifespan (Days) ± SEM	n=
**Male**			
Control	58.5	60.08 ± 1.76	50
Ratio 1:1	62.5	61.10 ± 1.37	50
Control	60	59.76 ± 1.58	50
Ratio 1:3	69	68.30 ± 1.36	50
Control	59	58.35 ± 2.44	20
Ratio 1:9	68.5	71.80 ± 2.81	20
Control	62	62.84 ± 1.82	50
Ratio 3:1	63	63.26 ± 1.47	50
**Female**			
Control	61.5	62.40 ± 1.57	50
Ratio 1:1	68	64.88 ± 1.38	50
Control	57	57.74 ± 1.39	50
Ratio 1:3	71	72.08 ± 1.49	50
Control	60	61.20 ± 2.72	20
Ratio 1:9	72	71.50 ± 2.19	20
Control	63	64.48 ± 1.26	50
Ratio 3:1	67	67.20 ± 1.58	50

Supplementary Figure 1

g

	Median lifespan (Days)	Mean Lifespan (Days) ± SEM	n=
**Male**			
Control	60	59.76 ± 1.58	50
Aged (40D) Flies + Fresh Young Flies	60.5	61.46 ± 1.54	50
**Female**			
Control	57	57.74 ± 1.39	50
Aged (40D) Flies + Fresh Young Flies	67.5	67.46 ± 1.22	50

Supplementary Figure 2

	Median lifespan (Days)	Mean Lifespan (Days) ± SEM	n=
**Male**			
Control	57	55.24 ± 2.18	49
Co-housed Aged (30D) Flies	57	57.22 ± 2.19	49
**Female**			
Control	61	59.56 ± 1.48	50
Co-housed Aged (30D) Flies	64	64.86 ± 1.88	50

	Median lifespan (Days)	Mean Lifespan (Days) ± SEM	n=
**Male**			
Control	61	62.40 ± 1.36	50
Co-housed Aged (50D) Flies	64	64.08 ± 1.19	50
**Female**			
Control	61.5	63.90 ± 1.42	50
Co-housed Aged (50D) Flies	70.5	71.24 ± 1.71	50

Supplementary Figure 3

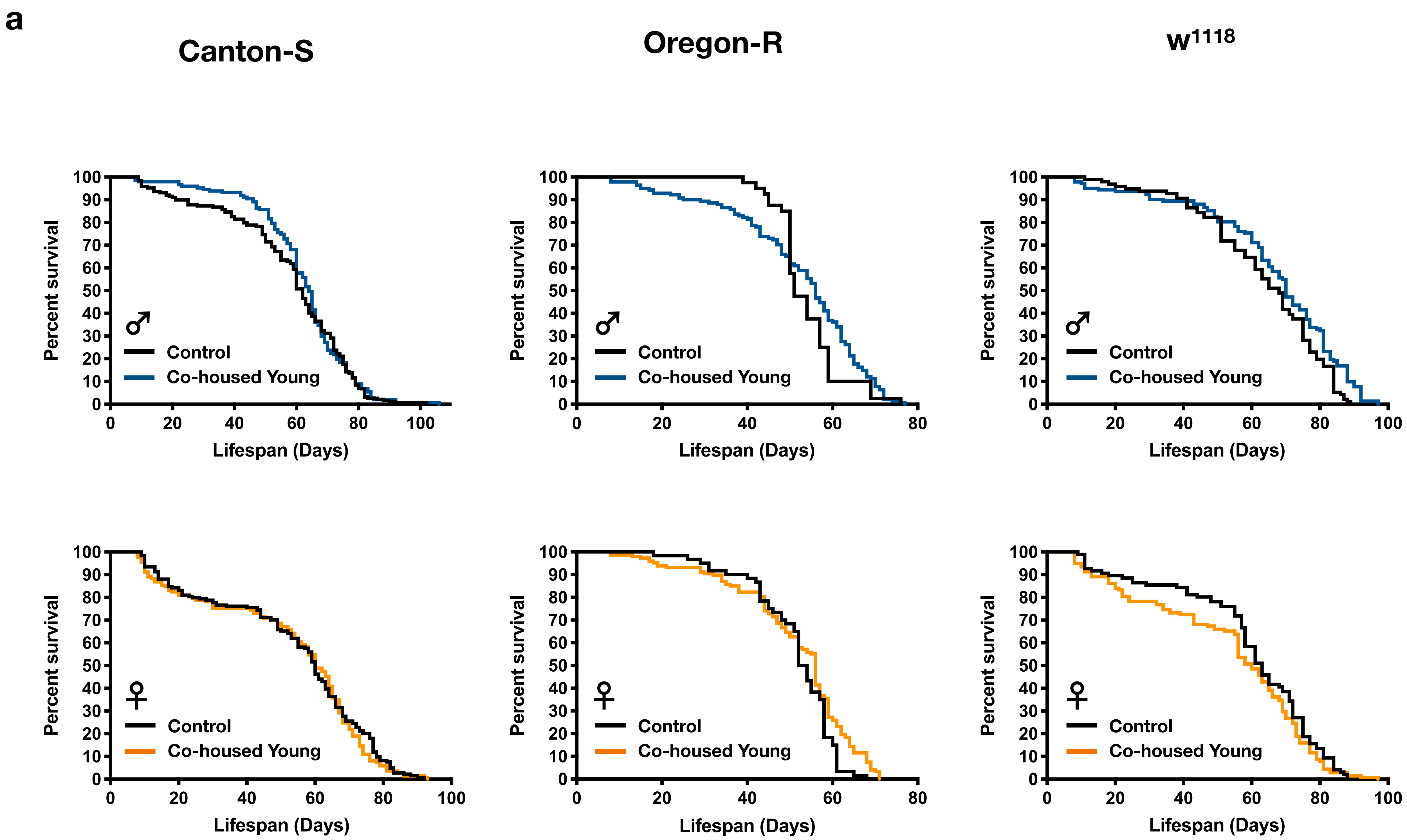

Supplementary Figure 3

b

Canton-S	Median Lifespan (Days)	Mean Lifespan (Days) ± SEM	n=
**Male**			
Control	62	57.76 ± 1.46	189
Co-housed Young Flies	64	61.97 ± 1.29	147
**Female**			
Control	60	53.55 ± 1.71	184
Co-housed Young Flies	60	52.72 ± 2.01	137

Oregon-R	Median Lifespan	Mean Lifespan (Days) ± SEM	n=
**Male**			
Control	51	53.88 ± 1.20	40
Co-housed Young Flies	56	52.18 ± 1.34	141
**Female**			
Control	53	51.22 ± 1.29	60
Co-housed Young Flies	56	51.56 ± 1.16	147

w^{1118}	Median Lifespan	Mean Lifespan (Days) ± SEM	n=
**Male**			
Control	68	63.51 ± 1.85	96
Co-housed Young Flies	70	66.71 ± 1.77	142
**Female**			
Control	63	59.09 ± 2.17	96
Co-housed Young Flies	60	53.46 ± 2.02	138

Supplementary Figure 3

c

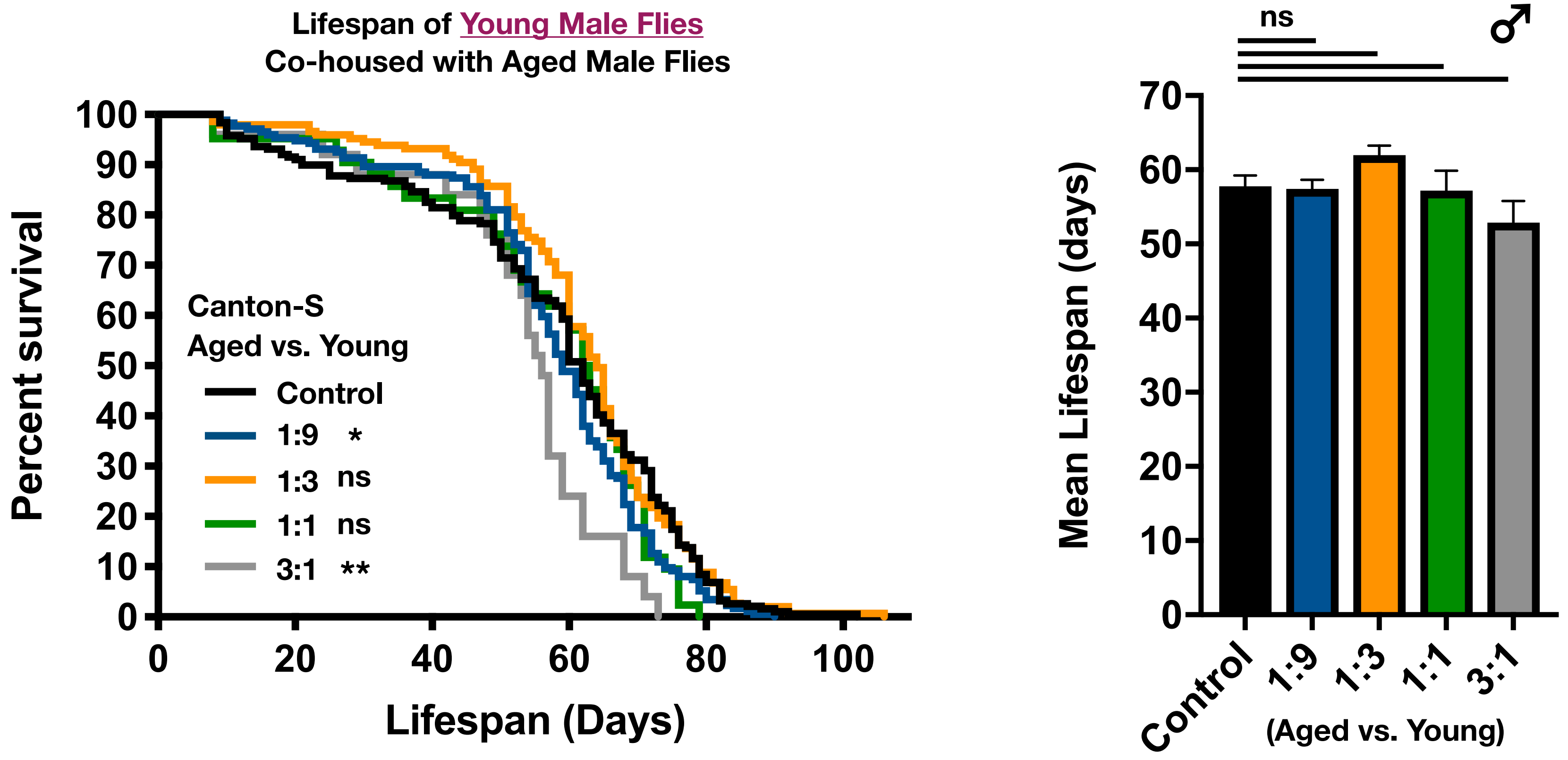

d

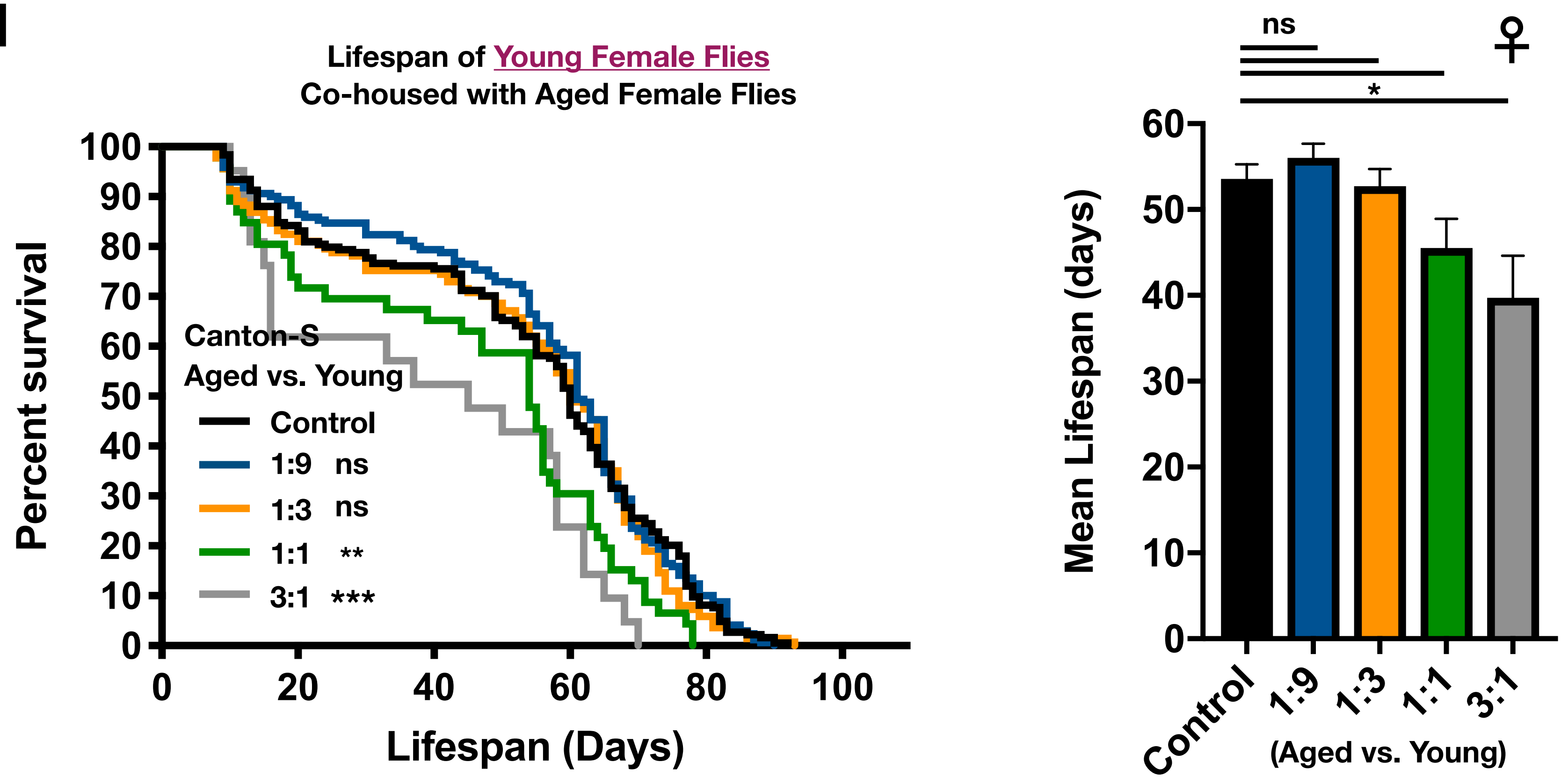

Supplementary Figure 3

e

	Median lifespan (Days)	Mean Lifespan (Days) ± SEM	n=
**Male**			
Control	62	57.76 ± 1.46	189
1:9	59	57.45 ± 1.23	174
1:3	64	61.97 ± 1.29	147
1:1	62.5	57.19 ± 2.72	42
3:1	56	52.88 ± 2.92	25
**Female**			
Control	60	53.55 ± 1.71	184
1:9	61	56.00 ± 1.67	170
1:3	60	52.72 ± 2.01	137
1:1	54	45.52 ± 3.39	46
3:1	45	39.71 ± 4.93	21

Supplementary Figure 4

	Median lifespan (Days)	Mean Lifespan (Days) ± SEM	n=
**Male**			
Control	60	59.76 ± 1.58	50
Aged + 1d	69	68.30 ± 1.36	50
Aged + 10d	61	60.08 ± 1.16	50
Aged + 20d	62	61.94 ± 1.29	50
Aged + 30d	63	62.26 ± 1.23	50
**Female**			
Control	57	57.74 ± 1.39	50
Aged + 1d	71	72.08 ± 1.49	50
Aged + 10d	61.5	65.54 ± 1.42	50
Aged + 20d	63	63.50 ± 1.13	50
Aged + 30d	67	64.46 ± 1.62	50

Supplementary Figure 5

a

	Median lifespan (Days)	Mean Lifespan (Days) ± SEM	n=
**Male**			
w[1118]	65	61.21 ± 2.11	100
or83b (-/-)	66	61.44 ± 1.65	100
ppk23 (-/-)	60	62.96 ± 0.95	118
**Female**			
w[1118]	61	56.97 ± 2.33	100
or83b (-/-)	82	74.44 ± 2.20	96
ppk23 (-/-)	77	77.16 ± 0.91	119

b

or83b (-/-)	Median lifespan (Days)	Mean Lifespan (Days) ± SEM	n=
**Male**			
Control	70	69.72 ± 1.09	25
co-housed 1d-old *w*[1118] flies (1:3)	72	73.68 ± 1.48	25
co-housed 1d-old *w*[1118] flies (1:9)	75	75.35 ± 0.98	26
**Female**			
Control	80	79.76 ± 1.73	25
co-housed 1d-old *w*[1118] flies (1:3)	83	83.84 ± 1.54	25
co-housed 1d-old *w*[1118] flies (1:9)	88	87.35 ± 1.25	26

ppk23 (-/-)	Median lifespan (Days)	Mean Lifespan (Days) ± SEM	n=
**Male**			
Control	74	75.28 ± 1.92	25
co-housed 1d-old *w*[1118] flies (1:3)	77	78.88 ± 1.84	25
co-housed 1d-old *w*[1118] flies (1:9)	81.5	82.42 ± 1.12	26
**Female**			
Control	85	82.60 ± 1.73	25
co-housed 1d-old *w*[1118] flies (1:3)	84	85.32 ± 1.64	25
co-housed 1d-old *w*[1118] flies (1:9)	88.5	89.62 ± 1.41	26

Supplementary Figure 6

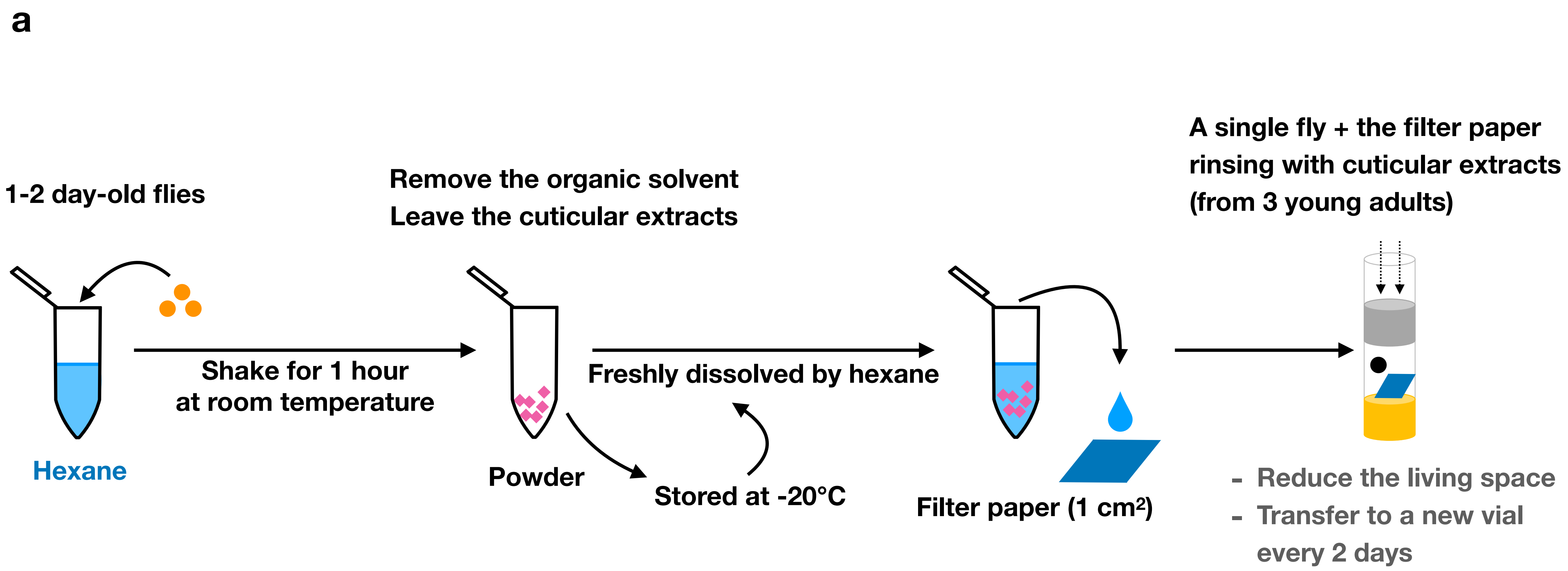

Supplementary Figure 6

b

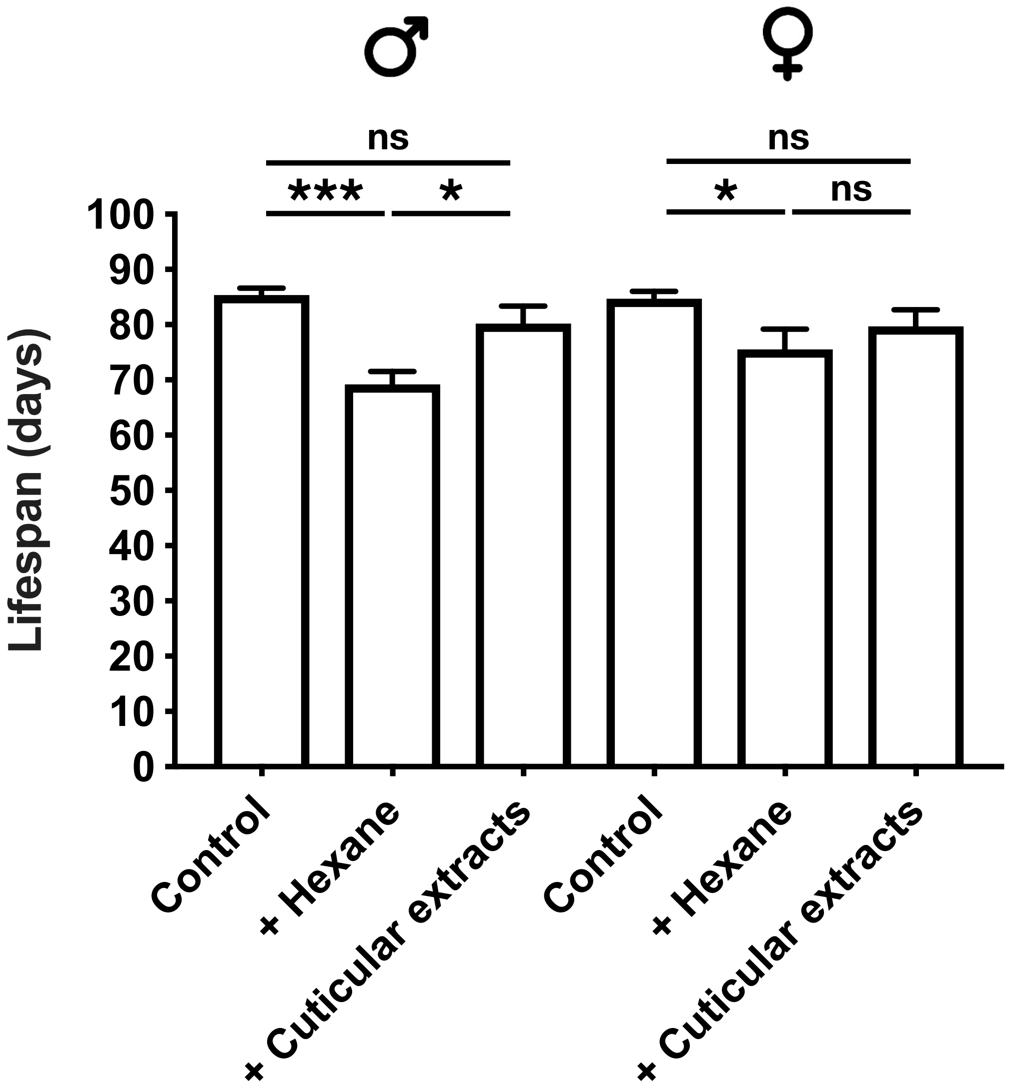

Mean Lifespan (Days) ± SEM	Male	n=
Control	85.4 ± 1.20	10
Hexane	69.1 ± 2.39	10
Cuticular extracts	80.2 ± 3.18	10

Mean Lifespan (Days) ± SEM	Female	n=
Control	84.7 ± 1.36	10
Hexane	75.5 ± 3.67	10
Cuticular extracts	79.7 ± 2.99	10

Supplementary Figure 7

Aged Flies Co-housed with	Median lifespan (Days)	Mean Lifespan (Days) ± SEM	n=
**Male**			
Young Control	69	68.30 ± 1.36	50
OK72>FASN^{CG3524} RNAi	58	57.56 ± 1.53	25
OK72>Cyp4g1 RNAi	56	55.32 ± 0.96	25
**Female**			
Young Control	60	72.08 ± 1.49	50
OK72>FASN^{CG3524} RNAi	57	61.28 ± 1.23	25
OK72>Cyp4g1 RNAi	61.5	59.28 ± 0.91	25

Raw Images of Western Blots

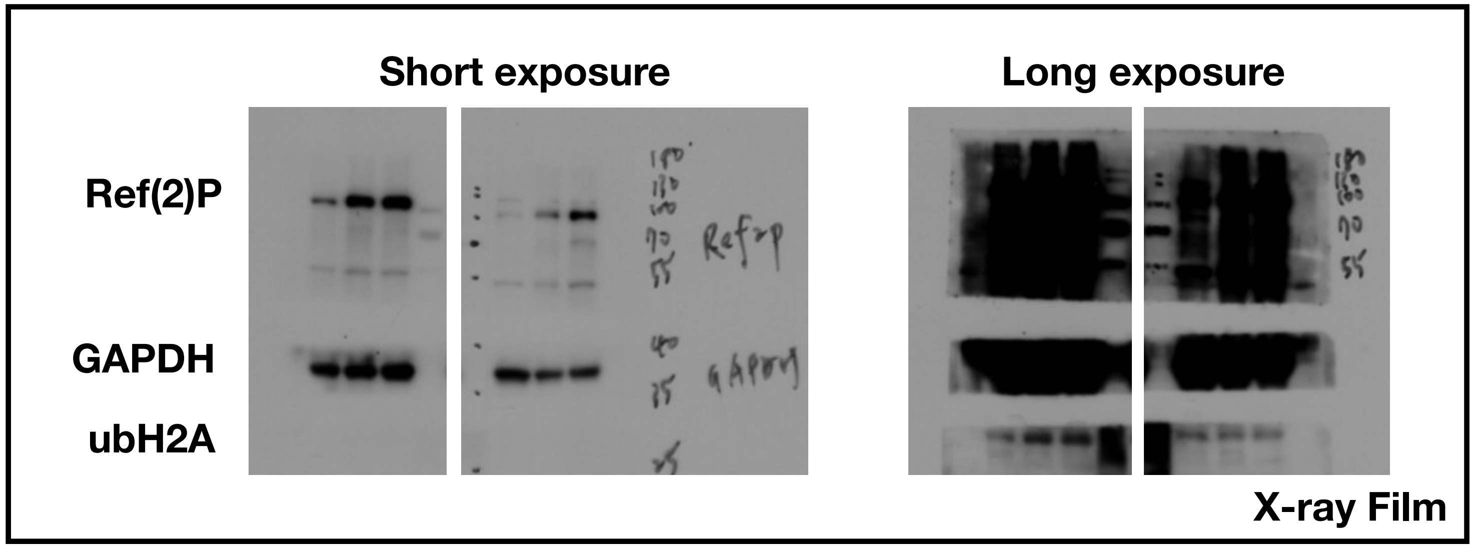

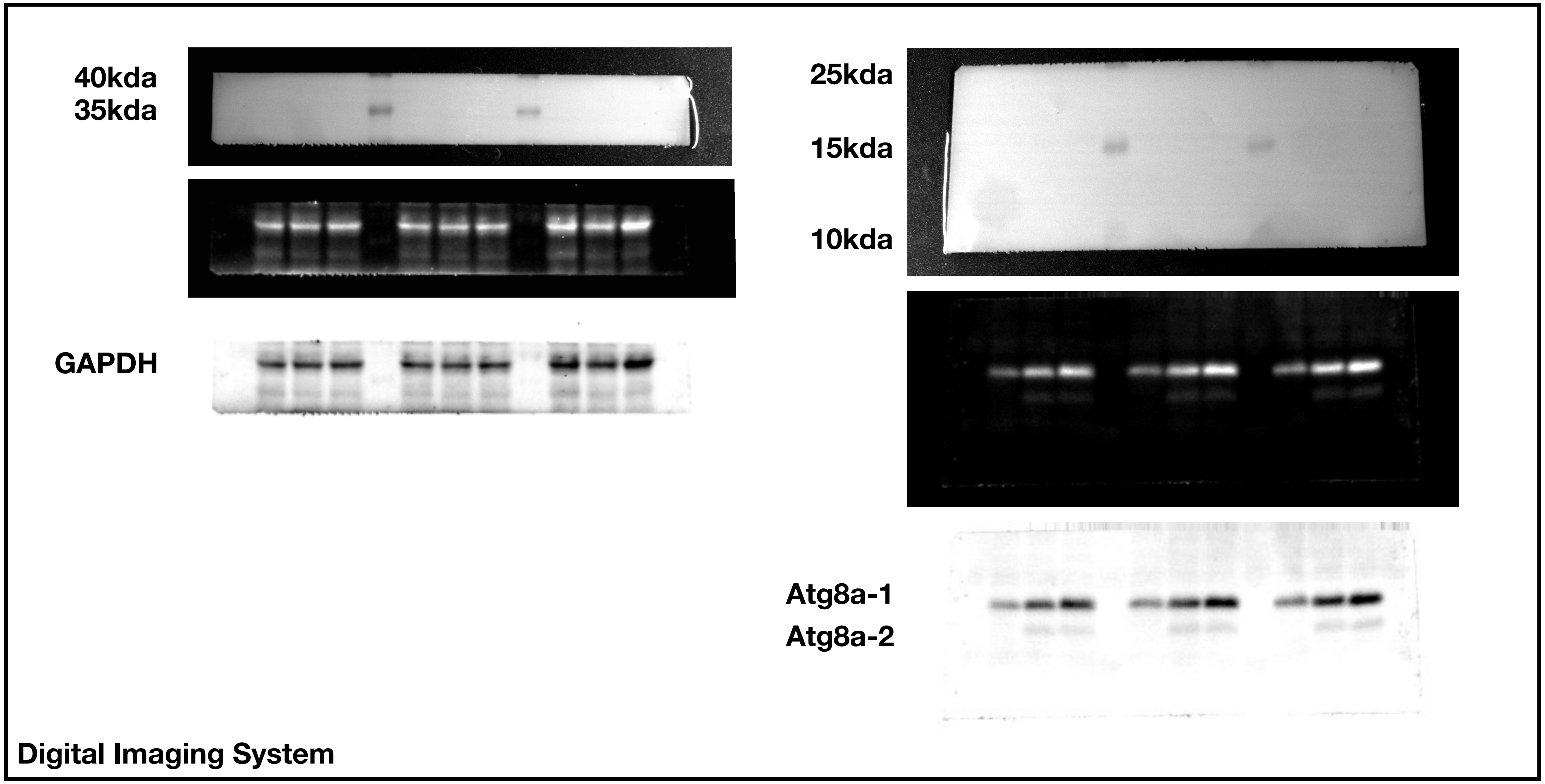

Supplement: Supplementary file 1 — Supplementary Information [file 41514_2021_73_MOESM1_ESM.pdf]
